# Supplementary material for: Platelets reduce anoikis and promote metastasis by activating YAP1 signaling
Source: Nat Commun. 2017 Aug 21;8:310. doi: 10.1038/s41467-017-00411-z (PMC5566477; doi:10.1038/s41467-017-00411-z)
Supplement: Supplementary file 1 — Supplementary Information [file 41467_2017_411_MOESM1_ESM.pdf]

File Name: Supplementary Information
Description: Supplementary Figures and Supplementary Tables

File Name: Supplementary Data 1
Description: RPPA log2 transformed data in HeyA8 co-incubated with or without platelets or platelets alone.

File Name: Peer Review File
Description:

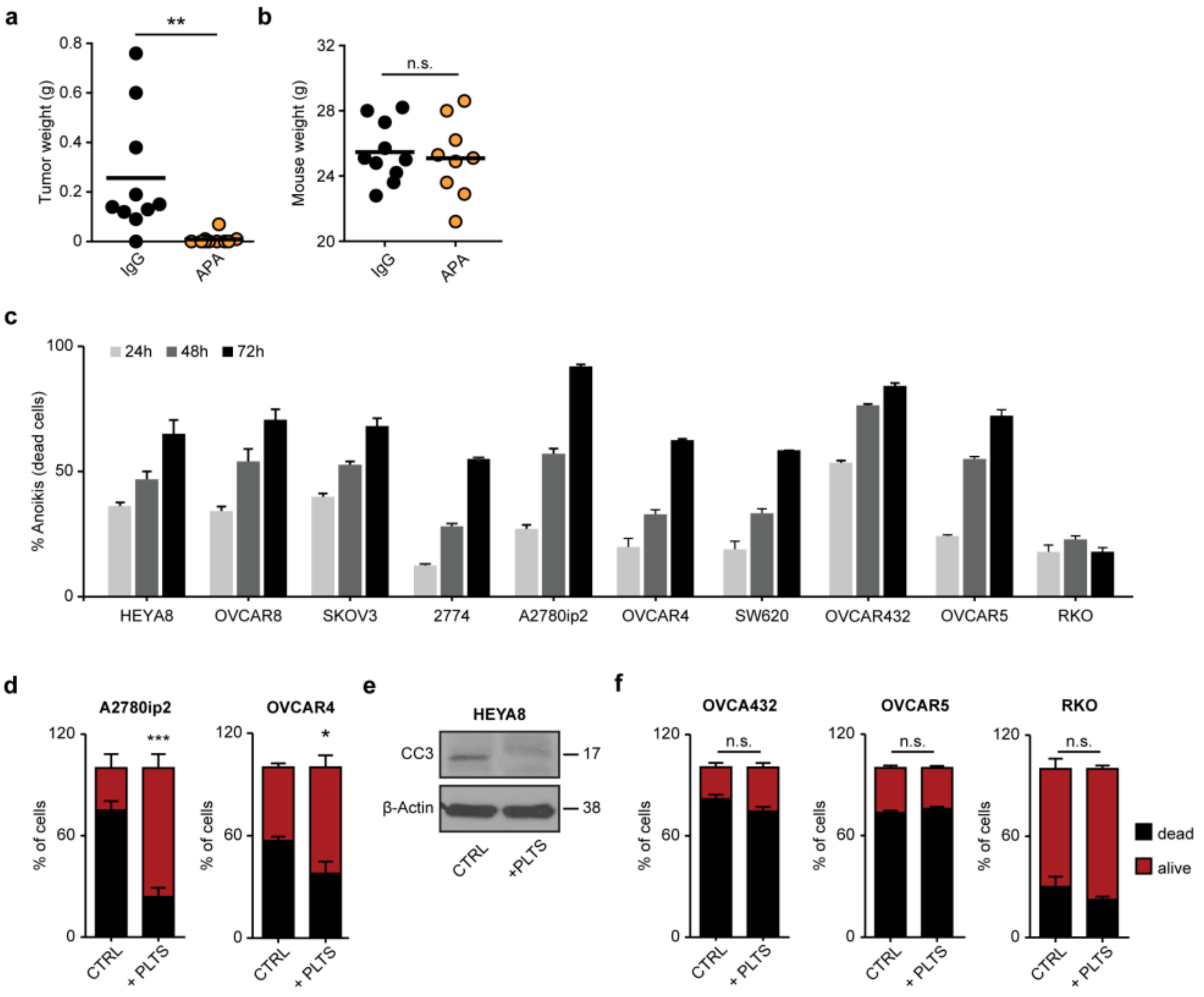

Supplementary Fig. 1 | Platelets facilitate metastasis in vivo and reduce anoikis. (a) Aggregate tumor weight (in g) of SW620 liver metastatic nodules in control and APA group (two-sided t-test, n=10). **(b)** Average mouse weight in control and APA group (two-sided t-test, n=10). **(c)** Average (± SEM) percentage of SYTOX® Red positive ovarian and colon cancer cells after 24, 48 and 72 hours growth under low attachment conditions. **(d)** Bar graphs representing the percentage of dead (SYTOX® Red positive, black) and living (SYTOX® Red negative, red) A2780ip and OVCAR4 cells co-incubated with or without 100 x 10^6 platelets for 72 hours under low attachment conditions (two-sided t-test, n=3). **(e)** Western blot analysis of cleaved caspase 3 in HeyA8 with or without platelet co-incubation for 72 hours under low attachment conditions

(n=3). Beta-actin was used as a loading control. **(f)** Bar graphs representing the percentage of dead (SYTOX® Red positive, black) and living (SYTOX® Red negative, red) OVCA432, OVCAR5 and RKO cells co-incubated with or without 100 x 10^6 platelets for 72 hours under low attachment conditions (two-sided t-test, n=3). Bars and error bars represent mean values and the corresponding SEMs (* P < 0.05, ** P < 0.01, *** P < 0.001).

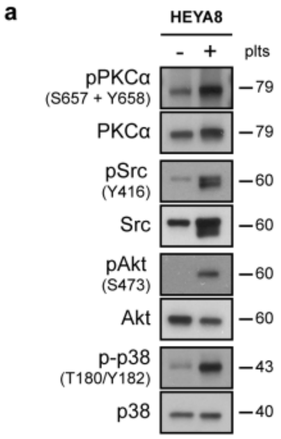

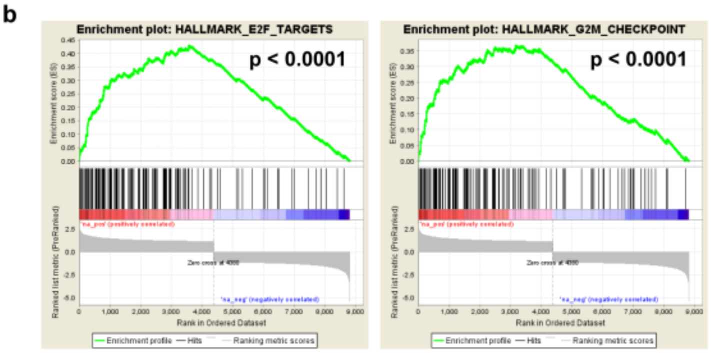

c

Enriched pathways (down)	p-value
HYPOXIA	< 0.0001
OXIDATIVE_PHOSPHORYLATION	< 0.0001
P53_PATHWAY	< 0.0001
TNFA_SIGNALING_VIA_NFKB	< 0.0001
DNA_REPAIR	< 0.0001
IL2_STAT5_SIGNALING	< 0.0001
APOPTOSIS	0.0049
KRAS_SIGNALING_UP	0.0148
COAGULATION	0.0261

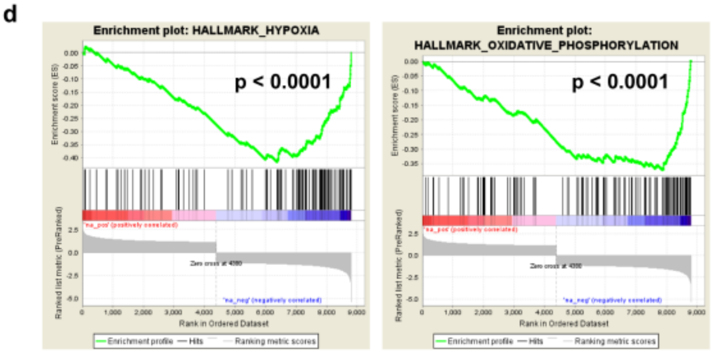

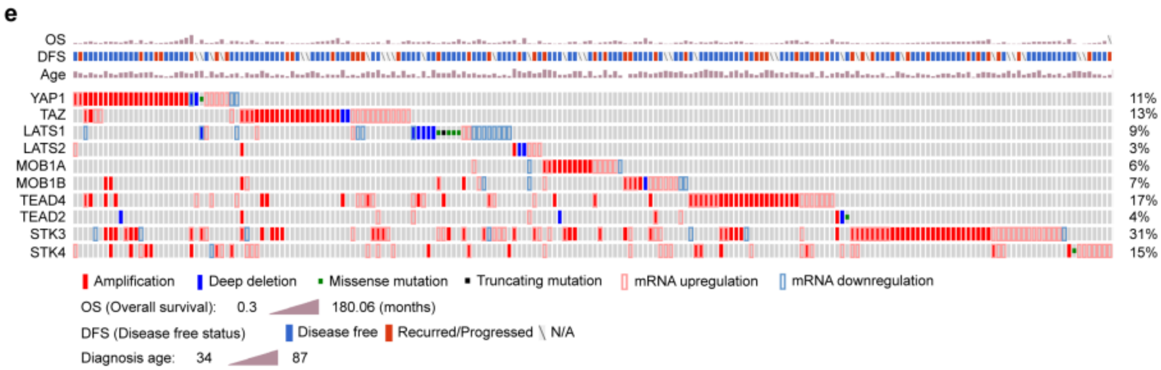

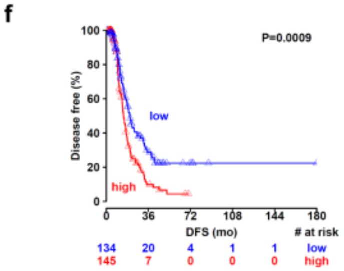

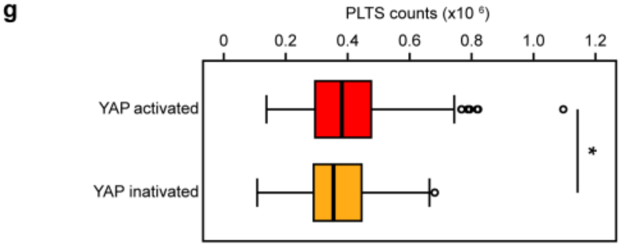

Supplementary Fig. 2 | Platelets induce a YAP1-specific gene signature in cancer cells. **(a)** Western blot validation of upregulated proteins after platelet co-incubation in HeyA8 as identified by RPPA analysis (n=3). **(b)** GSEA plots of enriched pathways in upregulated genes in HeyA8 after platelet co-incubation. **(c)** List of enriched pathways in downregulated genes in HeyA8 after platelet co-incubation and corresponding GSEA plots of top two **(d)**. **(e)** TCGA data analysis for YAP and Hippo pathway components in high-grade serous ovarian cancer samples (www.cbioportal.org). **(f)** Survival analysis of high-grade serous ovarian cancer patients with high and low protein expression of YAP. **(g)** Platelet counts in TCGA ovarian cancer tumors segregated by the prediction of a tumor YAP activation signature (two-sided t-test). Bars and error bars represent mean values and the corresponding SEMs. (* $P < 0.05$).

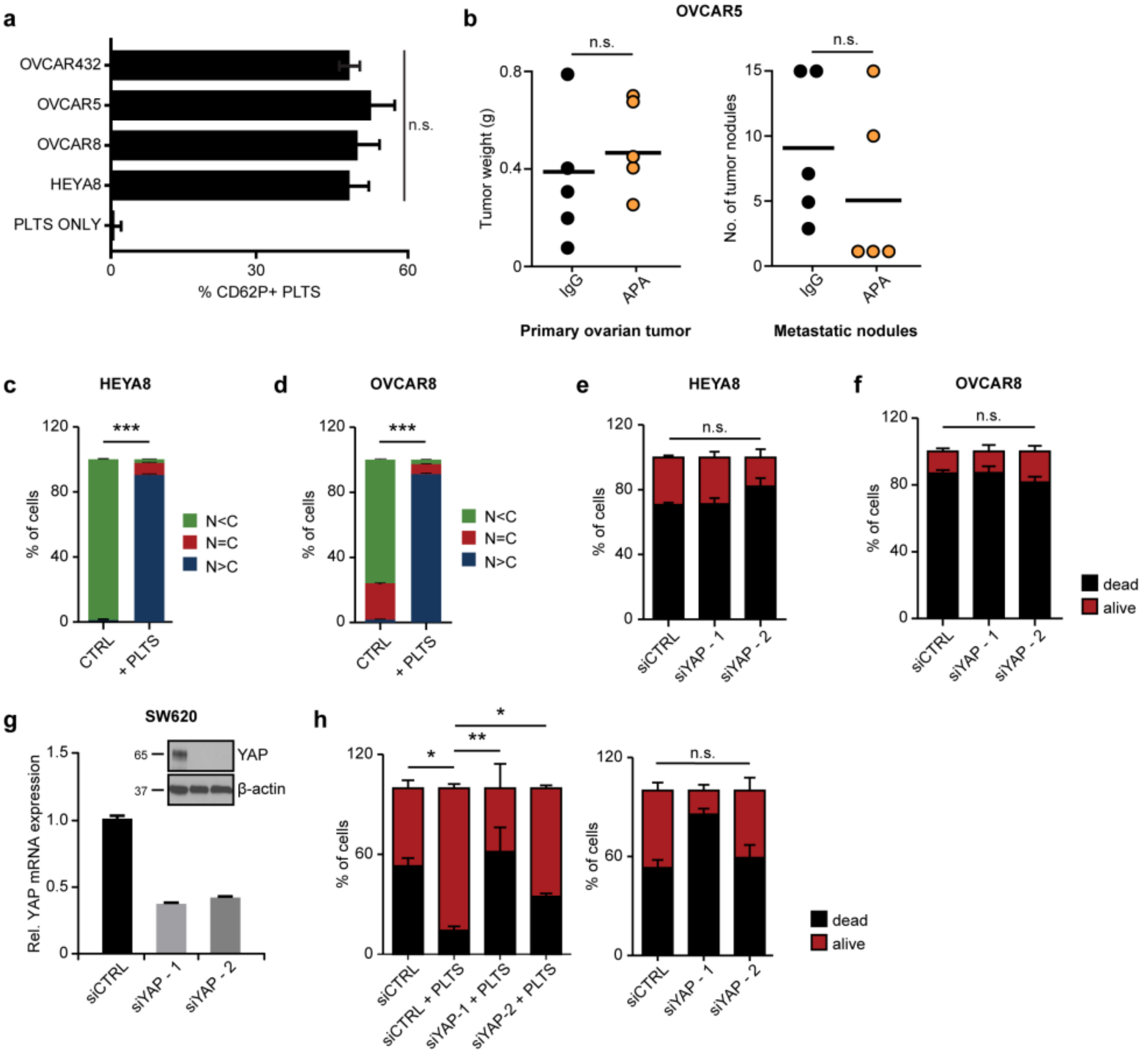

Supplementary Fig. 3 | YAP1 is activated by platelets and is indispensable for platelet-induced anoikis resistance. (a) Percentage of activated platelets as measured by surface expression of CD62P after co-incubation of platelets with ovarian cancer cell lines (n=3, one-way ANOVA followed by a Tukey's multiple comparison post-hoc test). **(b)** Tumor weight of primary ovarian tumor and number of metastatic nodules after intraovarian injection of OVCAR5 human ovarian cancer cell line and treatment with ctrl IgG or APA (n=5, two-sided t-test). **(c, d)** Quantification of nuclear versus cytoplasmic YAP in HeyA8 (c) and OVCAR8 (d) after co-incubation with platelets (n=3, two-sided t-test). **(e, f)** Percentage of dead (SYTOX® Red

positive, black) and living (SYTOX® Red negative, red) HeyA8 (e) or OVCAR8 (f) cells 96 hours after transfection of control and two different YAP siRNAs (n=3, two-sided t-test). **(g)** Validation of knockdown of YAP in SW620 cells on the protein and RNA level (n=3). **(h)** Percentage of dead (SYTOX® Red positive, black) and living (SYTOX® Red negative, red) SW620 cells 72 hours after addition of platelets and 96 hours after siRNA transfection (n=3, two-sided t-test). Bars and error bars represent mean values and the corresponding SEMs (* P < 0.05, ** P < 0.01, *** P < 0.001).

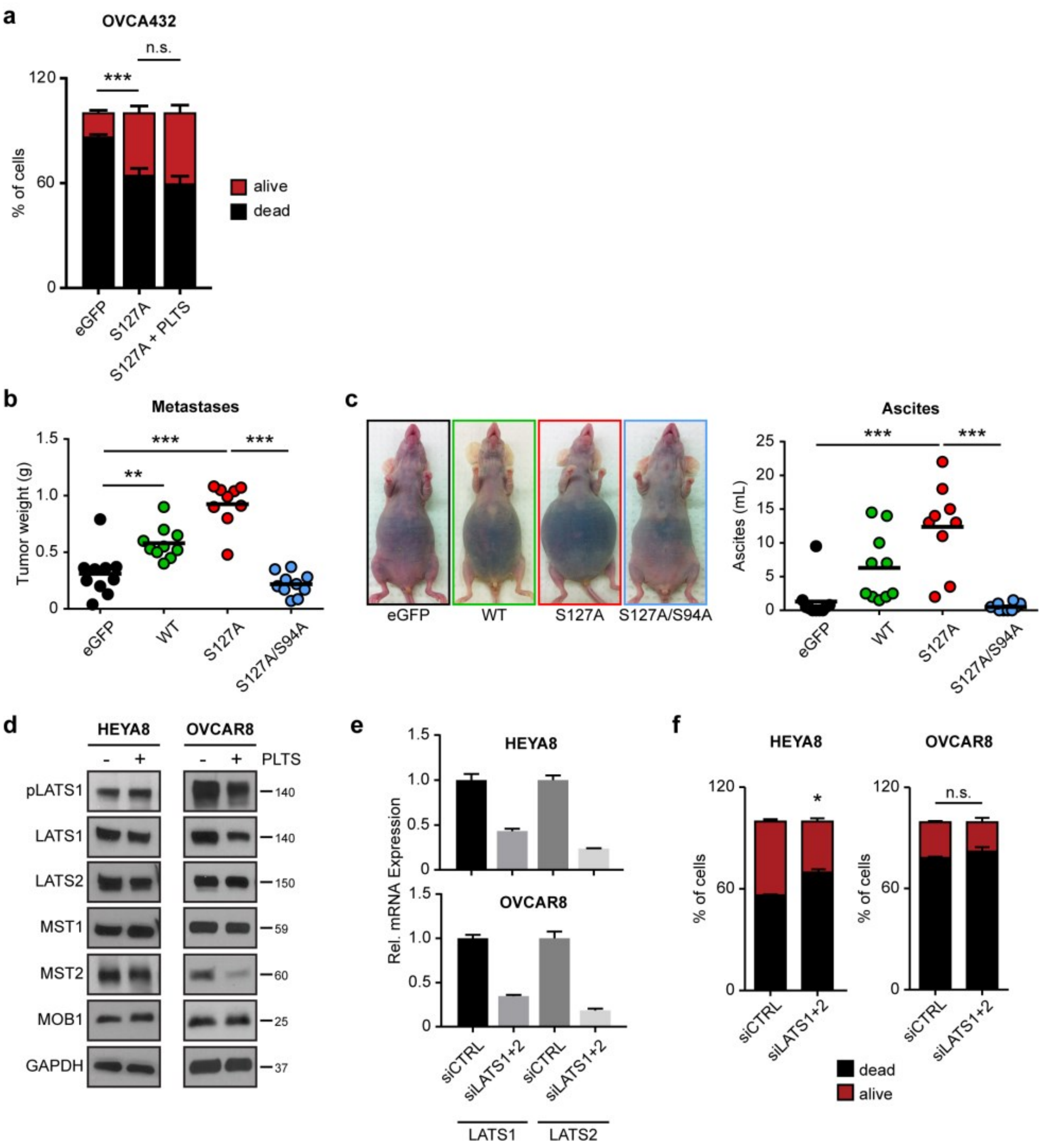

Supplementary Fig. 4 | Role of YAP overexpression and upstream Hippo pathway components in platelet-induced anoikis resistance. (a) Co-incubation of OVCA432 overexpressed with YAP^{S127A} with platelets and measurement of dead (SYTOX® Red positive, black) and living (SYTOX® Red negative, red, n=3, two-sided t-test). **(b)** Aggregate tumor weight

(in g) of metastatic nodules in indicated groups after intraovarian injection of OVCA432 cells (n=10, one-way ANOVA followed by a Tukey's multiple comparison post-hoc test). **(c)** Representative necropsy pictures and amount of ascites (in mL) in indicated groups after intraovarian injection of OVCA432 cells (n=10, one-way ANOVA followed by a Tukey's multiple comparison post-hoc test). **(d)** Western blot analysis of the Hippo pathway components LATS1, LATS2, MST1, MST2 and MOB1 in HeyA8 (left) and OVCAR8 (right) with or without platelet co-incubation for two hours (n=3). **(e)** Validation of LATS1 and LATS2 knockdown in HeyA8 and OVCAR8 cells 72 hours after siRNA transfection (n=3). **(f)** Bar graphs representing number of dead (SYTOX® Red positive, black) and living (SYTOX® Red negative, red) HeyA8 or OVCAR8 ovarian cancer cells after growing 72 hours in low attachment and 96 hours after siRNA transfection (n = 3, two-sided t-test). Bars and error bars represent mean values and the corresponding SEMs (* $P < 0.05$, ** $P < 0.01$, *** $P < 0.001$, n.s. = non-significant).

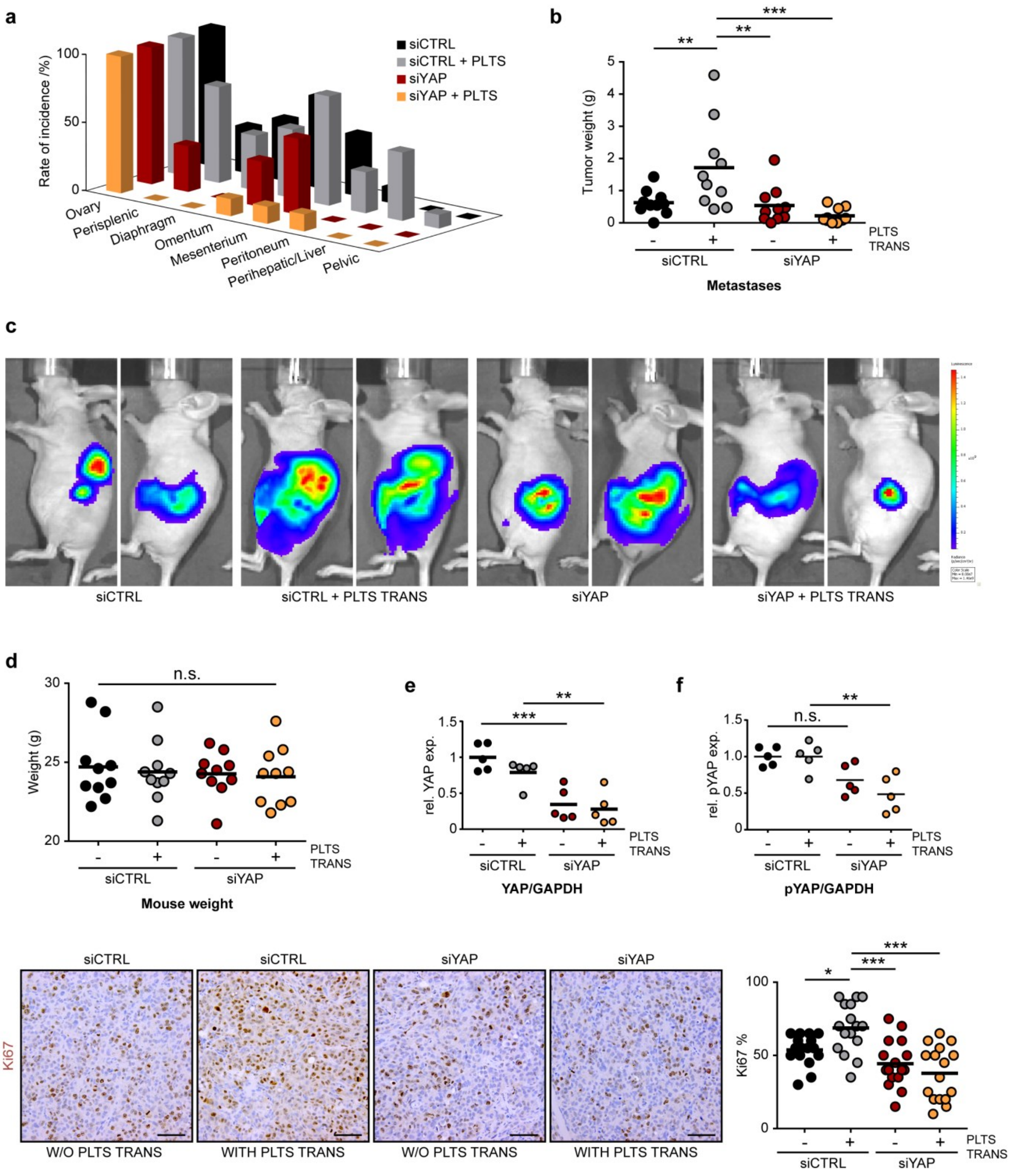

Supplementary Fig. 5 | Inhibition of YAP1 in vivo impedes thrombocytosis-enhanced metastasis. (a) Location and rate of occurrence (%) of metastatic nodules in respective groups. **(b-d)** Mean aggregate tumor weight of metastatic nodules (b), representative bioluminescence

imaging pictures (c) and average body weight (d) of mice injected with HeyA8 human ovarian cancer cells into the ovary and treated with either control or YAP siRNA with or without platelet transfusions twice weekly for the duration of the experiment (n=10, one-way ANOVA followed by a Tukey's multiple comparison post-hoc test). **(e, f)** Densitometric quantification of YAP and pYAP expression relative to GAPDH (western blots shown in Fig. 5f) in whole tumor lysates in respective groups (n=5, one-way ANOVA followed by a Tukey's multiple comparison post-hoc test). **(g)** Immunohistochemical stainings for Ki67 and quantification of the percentage of Ki67-positive cells per high power field (HPF) in tumors treated with control (siCTRL) or YAP (siYAP) siRNA with or without platelet transfusions twice weekly. Representative immunohistochemical images after processing of tumors from 7 individual mice (one-way ANOVA followed by a Tukey's multiple comparison post-hoc test, Scale bars = 50 µm). Bars and error bars represent mean values and the corresponding SEMs (* $P < 0.05$, ** $P < 0.01$, *** $P < 0.001$, n.s. = non-significant).

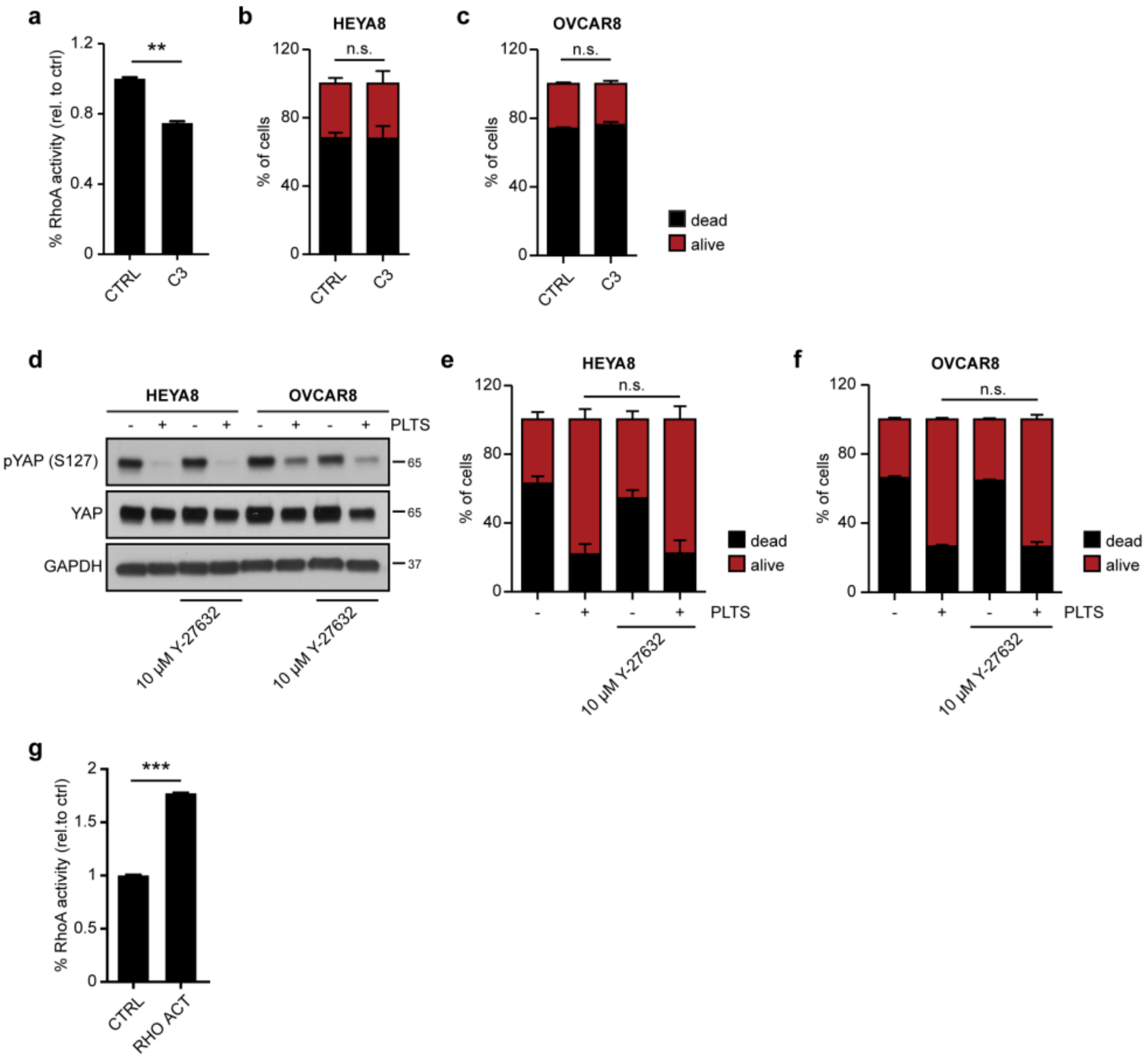

Supplementary Fig. 6 | Regulation of YAP1 activity by RhoA-MYPT1-PP1 axis controls anoikis. (a) Percentage of Rho activation levels after treatment of HeyA8 cells with 1 µg ml^{-1} Rho inhibitor C3 transferase for 4 hours under low attachment conditions (n=3, two-sided t-test). **(b, c)** Percentage of dead (SYTOX® Red positive, black) and living (SYTOX® Red negative, red) HeyA8 (b) or OVCAR8 (c) either untreated or treated with 1 µg ml^{-1} of the Rho inhibitor C3 transferase (n=3, two-sided t-test). **(d)** Western blot analysis of phosphorylated (S127) and total YAP in HeyA8 and OVCAR8 co-incubated with platelets for two hours under low attachment conditions with or without the treatment of 10 µM Y-27632. GAPDH was used as a loading

control (n=3). **(e, f)** Percentage of dead (SYTOX® Red positive, black) and living (SYTOX® Red negative, red) HeyA8 (e) or OVCAR8 (f) cells treated with 10 µM Y-27632 with or without platelet co-incubation for 72 hours (n=3, one-way ANOVA followed by a Tukey's multiple comparison post-hoc test). **(g)** Percentage of Rho activation levels after treatment of HeyA8 cells with 1 U ml^{-1} Rho activator for 2 hours under low attachment conditions (n=3, two-sided t-test). Bars and error bars represent mean values and the corresponding SEMs (* $P < 0.05$, ** $P < 0.01$, *** $P < 0.001$, n.s. = non-significant).

Supplementary Fig. 7 | Uncropped scans of blots

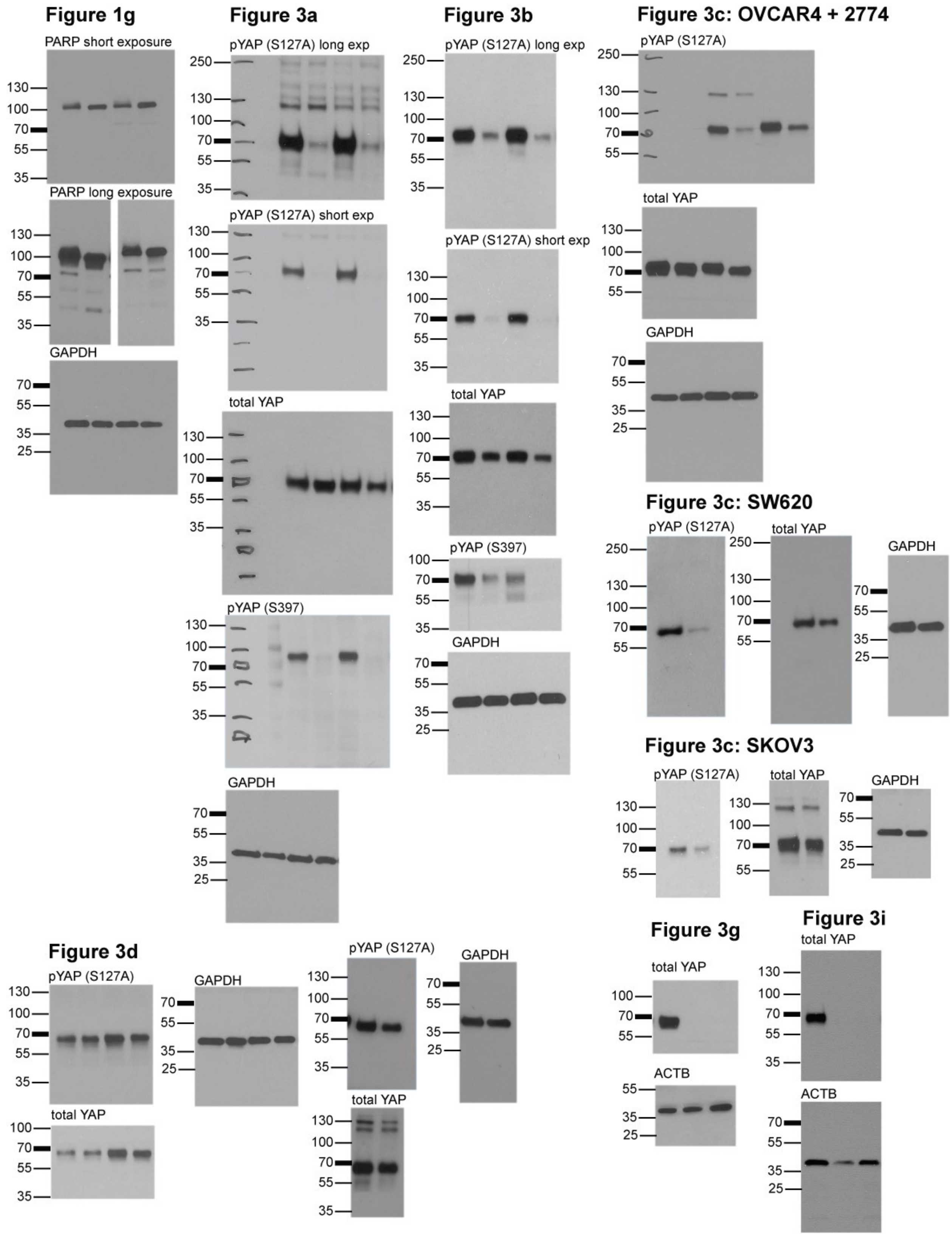

Supplementary Fig. 7 (continued) | Uncropped scans of blots

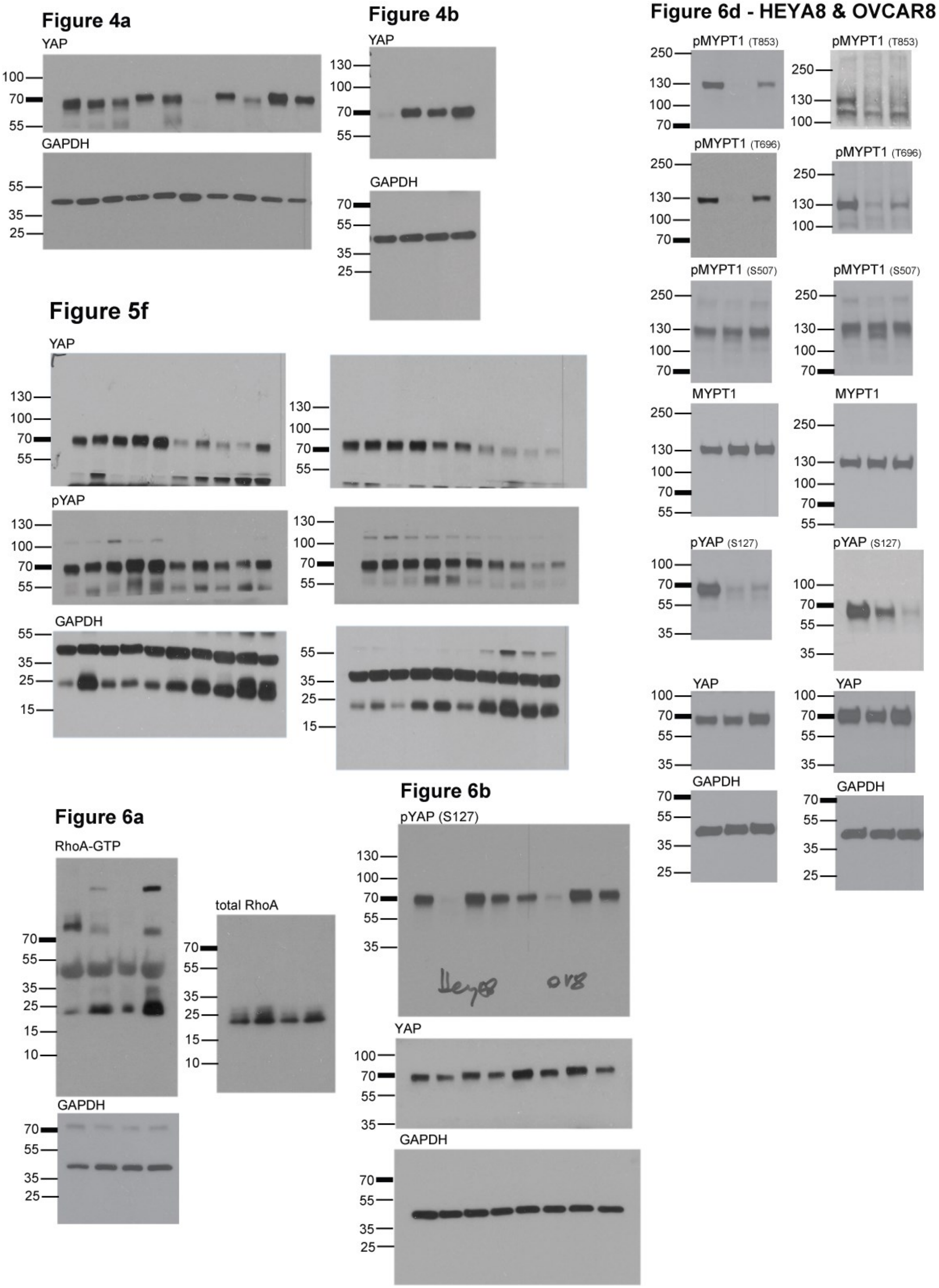

Supplementary Fig. 7 (continued) | Uncropped scans of blots

Figure 6e - HEYA8

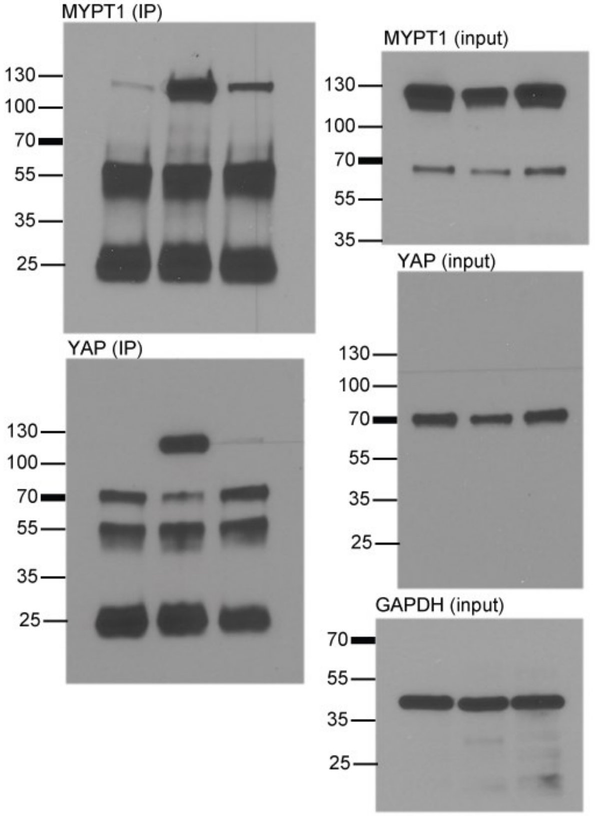

Figure 6e- OVCAR8

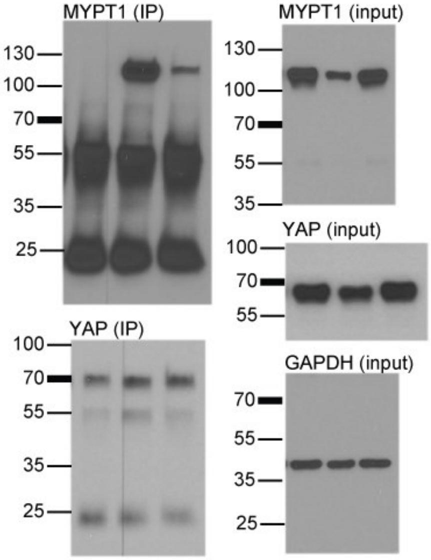

Figure 6f

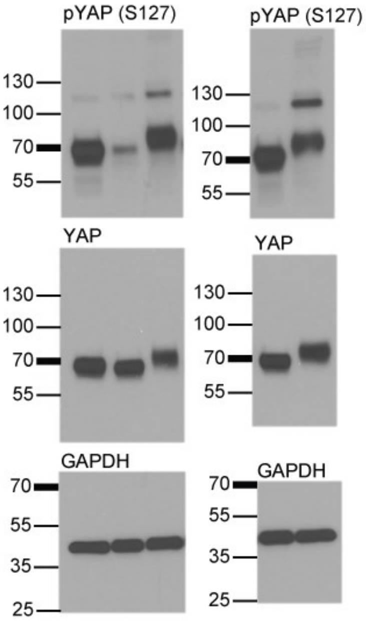

Figure 6g

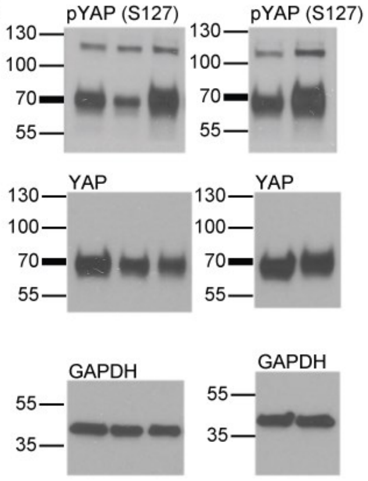

Supplementary Fig. 7 (continued) | Uncropped scans of blots

Suppl Fig. 1e

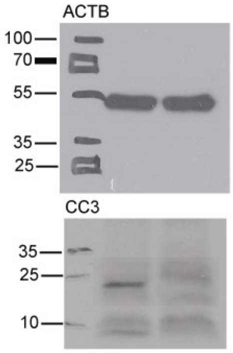

Suppl Fig. 2a

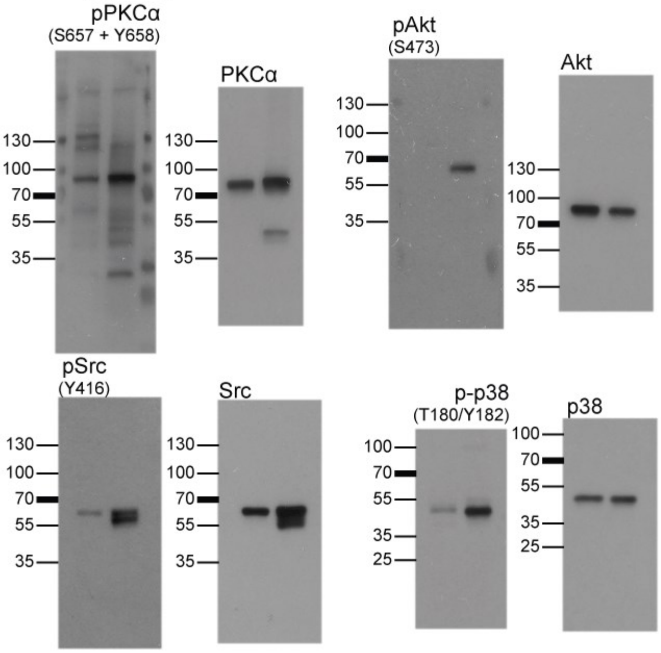

Suppl Fig. 3g

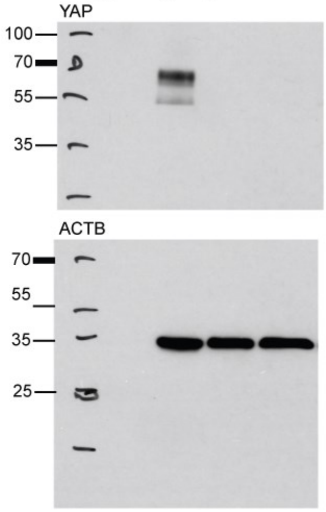

Suppl Fig. 4d
HEYA8

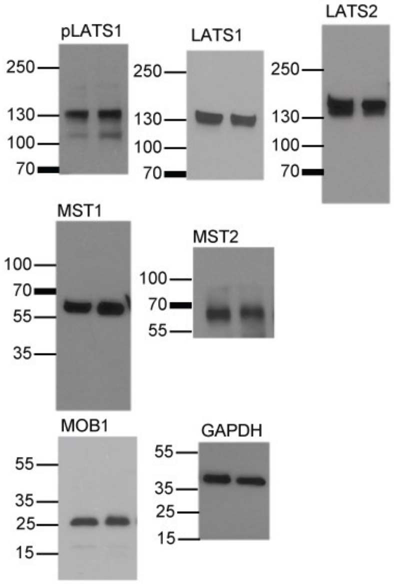

Suppl Fig. 4d
OVCAR8

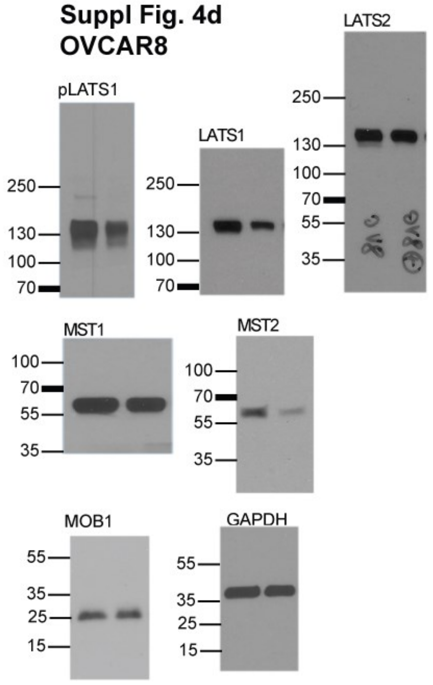

Suppl Fig. 6d

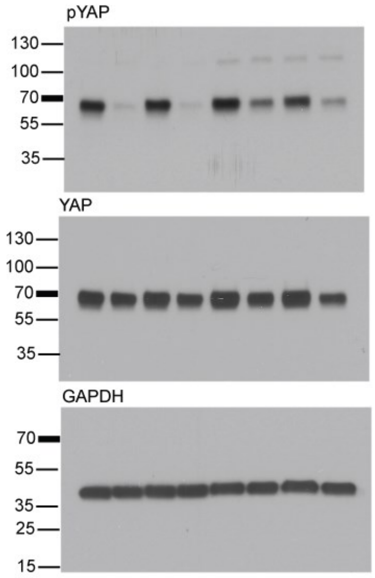

Supplementary Table 1: Demographic information on ovarian cancer patients whose tumors were used for YAP immunohistochemistry

**Age**	Median 64 (range 46-82)
**Stage**	IIIB – 1<br>IIIC – 16<br>IV – 4
**Grade**	High grade – 21
**Platelet counts**	Median 463,000 (range 144,000 – 683,000)

Supplementary Table 2: Sequence of qRT-PCR primers

Gene name	forward (5'-3')	reverse (5'-3')
18S	CGCCGCTAGAGGTGAAATTC	TTGGCAAATGCTTTCGCTC
YAP1	CACAGCTCAGCATCTTCGAC	GCCATGTTGTTGTCTGATCG
LATS1	CTGTCCAGGAGCTCTGCTCT	ATGTAGCCCACACGAAGGAC
LATS2	ACAAGATGGGCTTCATCCAC	CATGGCTCCCTTTCTGGTAA
DDIT4	GGTGTGACATCCAGAGAGCA	TACACAAACCACCTCCACGA
NDRG1	GTCGAGGCTAGAGGCATTTG	CCTGCTTTTGCTGCACATTA
CDC20	GAGGTGCAGCTATGGGATGT	ACATCATGGTGGTGGATGTG
CTGF	CCTGCAGGCTAGAGAAGCAG	TGGAGATTTTGGGAGTACGG
MCM5	GACTTCATGCCCACCATCTT	TCACGTGCAGAGTGATGACA
MCM6	GTGCGGGATCAAGTTGCTAT	GTCCACTGATTGGGTTTGCT
MCM10	CTTCACCTCCAGATCCCAAA	TTGAGTCGTTTCCCCAGAAC
